# Low-Rank Regularization for Learning Gene Expression Programs

**DOI:** 10.1371/journal.pone.0082146

**Published:** 2013-12-17

**Authors:** Guibo Ye, Mengfan Tang, Jian-Feng Cai, Qing Nie, Xiaohui Xie

**Affiliations:** 1 Department of Computer Science, University of California Irvine, Irvine, California, United States of America; 2 Department of Mathematics, University of California Irvine, Irvine, California, United States of America; 3 Department of Mathematics, University of Iowa, Iowa City, Iowa, United States of America; 4 Center for Complex Biological Systems, University of California Irvine, Irvine, California, United States of America; Manchester University, United Kingdom

## Abstract

Learning gene expression programs directly from a set of observations is challenging due to the complexity of gene regulation, high noise of experimental measurements, and insufficient number of experimental measurements. Imposing additional constraints with strong and biologically motivated regularizations is critical in developing reliable and effective algorithms for inferring gene expression programs. Here we propose a new form of regulation that constrains the number of independent connectivity patterns between regulators and targets, motivated by the modular design of gene regulatory programs and the belief that the total number of independent regulatory modules should be small. We formulate a multi-target linear regression framework to incorporate this type of regulation, in which the number of independent connectivity patterns is expressed as the rank of the connectivity matrix between regulators and targets. We then generalize the linear framework to nonlinear cases, and prove that the generalized low-rank regularization model is still convex. Efficient algorithms are derived to solve both the linear and nonlinear low-rank regularized problems. Finally, we test the algorithms on three gene expression datasets, and show that the low-rank regularization improves the accuracy of gene expression prediction in these three datasets.

## Introduction

Systematically discovering gene expression programs within cells is a fundamental goal in both basic and applied biomedical researches, and is crucial for elucidating factors determining cell types, controlling cellular states, or switching cells from healthy states to diseased ones. Although the total number of genes within an organism is usually large (e.g., 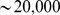 in humans), most of these genes are believed to be regulated by a much smaller subset of genes called regulators (e.g., transcription factors, signalling molecules, growth factors, etc.) A challenge in computational biology is how to use machine learning methods to automatically discover the mapping from regulators to target genes, thereby inferring the underlying regulatory programs, from a given set of observations [1].

More specifically, suppose we are given a set of observations 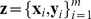, where 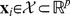 denotes the expression of 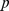 regulators and 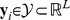 denotes the expression of 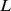 target genes in sample 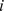. The goal of learning gene expression programs is to infer the mapping 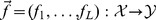 that fits the observation 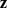, and to provide biological interpretations of the inferred mapping.

In addition to the purpose of uncovering gene regulatory mechanisms, the gene expression program learning problem arises recently also in a practical and applied setting in biotechnology development. High-throughput gene expression profiling using Affymetrix arrays typically costs 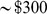 and 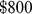 for human and mouse, respectively, which is still too expensive to be used in large-scale perturbation, drug or small molecule screening, which typically requires tens of thousands of or even millions of expression profiles [2]. This constraint has motivated the development and adoption of the Luminex bead technology, which is able to measure 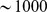 genes at a much lower cost (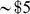 per profile). Because the gene expression is so highly correlated, scientists are proposing to use Luminex bead to measure 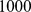 carefully chosen “landmark” genes and to computationally extrapolate all remaining ones. This strategy will be able to significantly cut the cost of expression profiling; however, it also calls for better and more efficient methods for target gene expression prediction.

Although the gene expression program learning problem formulated above fits into standard supervised learning, solving the problem is difficult for a number of reasons. First, the total number of parameters determining the mapping from regulators to target genes is typically much greater than the total number of observations. Secondly, the gene expression measurements based on high-throughput techniques are known to be highly noisy. These factors make the gene expression program inference highly challenging. A number of methods have been proposed for gene expression program learning, including methods based on probabilistic graphical models [3]–[5], information-theoretic approaches [6], [7], and ordinary differential equations (ODEs) [8], [9]. See [1], [10] for reviews of these and other approaches. However, the performance of these methods tend to be modest.

In this work, we formulate the gene expression program learning as a multi-target (more specifically 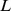-target) regression problem, and use Tikhonov regularization to constrain the space of the 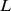-target mapping. Two main forms of regularization with biological motivations have been proposed in the literature: a) *sparsity* - each target is likely regulated by only a few regulators instead of all, and b) *modularity* - the expression program is organized into modules, each consisting of a certain combination of regulators, and the total number of independent modules should be small. The sparsity regularization is well recognized and widely used in gene expression program inference [9], [11], [12]. Some of these previous work are based on graphical models [4], [13], while others are based on regression. Within the regression framework, a common strategy of imposing sparsity is to use 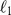 norm regularization on regression coefficients, similar to the framework of Lasso (least absolute shrinkage and selection operator) [11], [14].

The modularity regularization is much more difficult to handle and is the focus of this work. A popular approach is the probabilistic graphic model proposed by Segal et al. [15], which assigns target genes into different modules and constrains the genes within each module to be identically distributed, and models the regulatory program associated with each module using a rule-based decision tree. However, the Segal model is difficult to train, requiring long running time and only being able to find locally optimal solutions. In addition, the Segal model only captures the qualitative relationship between the regulators and targets since its main purpose is not on predicting the expression of target genes. Another approach taking the modularity structure into account is the SIMoNe proposed by Chiquet et al. [16]. SIMoNe models gene expression data using Gaussian graphical models, and imposes sparsity constraints on the inverse covariance matrix and introduces hidden nodes to the Gaussian graph to learn the network modularity. The primary goal of SIMoNe is to infer the gene regulatory networks in an unsupervised way without distinguishing regulator and target genes, which is different from our main objective.

Here we propose a new approach to incorporating the modularity constraint. We use the rank of the connectivity matrix between regulators and targets to represent the number of independent regulatory modules between them. The modularity regularization is then formulated as a low-rank constraint within a multi-target linear regression framework. The resulting model is convex, and we describe an efficient algorithm to find its globally optimal solution. We further show that the low-rank regularized regression problem can also be generalized to nonlinear cases, where we regularize the dimension of the hypothesis space of the 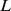-target regression function. We prove that the resulting nonlinear low-rank model is still convex and derive an efficient algorithm to solve it. Finally, we benchmark the performance of the low-rank regulation models on two real biological datasets, and show that the low-rank regulation technique consistently improve prediction accuracy in both cases when compared to the Lasso model.

## Methods

### Learning gene expression programs in linear space

We begin by introducing some notations. Let 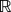 be the set of real numbers and 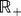 the subset of non-negative ones. Denote 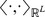 to be the inner product of 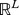. We assume that we have 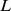 target genes and define 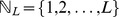. We further assume that for the 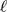-th target gene (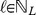), 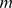 samples 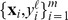 are available, where 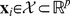 denotes the expression of 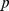 regulators and 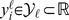 denotes the expression of the 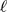-th target gene in sample 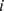. Let 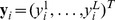 and 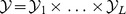. The goal of learning gene expression programs is to infer the mapping 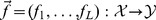 that fits the observation 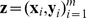, and to provide biological interpretations of the inferred mapping.

In this section, we assume that each target gene is linearly regulated by the regulators. That is, for each 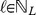, 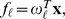 where 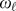 is a fixed vector of coefficients.

Next we describe two types of regularization that can be used for gene expression program learning: one is the *sparsity* regularization, which has already been widely used in the literature, and the second is the *low-rank* regularization, which has not been used in the gene expression program learning, although having recently become popular in other problem domains such as matrix completion, covariance matrix estimation, metric learning, etc [17]–[20].

### Sparsity regularization

Given the observation 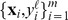, a natural way to infer 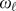 is to solve a least-square minimization problem:
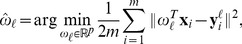
(1)where the norm is the 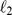 norm by default. However, for the gene expression program learning problem, the 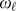 inferred by least-square minimization tends to be poor for a number of reasons: 1) the observations as measured by microarrays are usually very noisy, and 2) 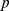 is usually much larger than 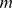, which can lead to overfitting. Various regularization techniques have been introduced to prevent overfitting including ridge regression [21] and Lasso [14]. Since each target gene is likely regulated by only a few regulators instead of all, a commonly used regularization technique in gene expression program learning is to impose an 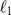-norm based sparsity regularization on 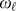 as in Lasso [9], [11], [12], [14]:
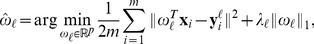
(2)where 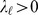 is a regularization parameter. We will call (2) the *Lasso model* in the following.

### Low-rank regularization

In the Lasso model, we treat each regulation function 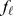 separately and learn them independently. However, it is well-known that the expression values of target genes are often highly correlated, and biologists believe that this high correlation is caused by sharing of regulatory programs among different genes. In addition, although there exist 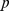 regulators in an organism, the number regulatory programs (called modules by biologists) active in a particular experimental setting is often much lower than 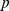. These considerations suggest that instead of learning each 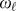 separately for each gene, we should learn all 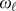
's jointly, and impose a new regularization on the dimension of the span of the 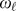
's.

Let 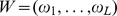 be a 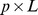 matrix with each column corresponding to one 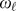. Constraining the dimension of the span of the 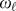
's is equivalent to regularizing the rank of 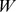, which motivates us to propose the following model to learn gene expression programs
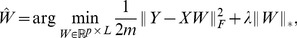
(3)where 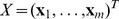, 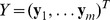 and 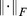 denotes the Frobenius norm for matrix. 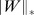 is the nuclear norm of matrix 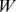, defined to be the sum of the singular values of 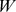. The nuclear norm is a convex function and is often used as a convex relaxation of the rank of 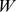 [22], [23]. Since nuclear norm is convex, model (3) is a convex optimization problem. We will call (3) the *linear low-rank model* in the following. The linear low-rank model has not been proposed for gene expression analysis, although it has appeared in other problem domains such as matrix completion, covariance matrix estimation, metric learning, etc [17]–[20].

### Low-rank regularization for learning gene expression programs in nonlinear space

Next we show that the low rank regularization can also be extended to learn nonlinear gene expression program. We start by proposing a low-rank regularization model in the Hilbert space, then prove that the model is a well-defined convex problem, and finally provide an algorithm to solve the model in the reproducing kernel Hilbert space (RKHS).

#### Low-rank model in Hilbert Space

We assume that each target gene is nonlinearly regulated by its regulators and the mapping 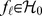, which is a Hilbert space. Furthermore, we assume that the mappings of different target genes are related to each other in such a way that 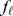 lies in a common low-dimensional subspace of 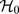. Note that the assumption of 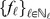 sharing a common subspace in Hilbert space is a natural generalization of the low-rank constraint in the linear case, where the weighting vectors 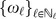 share a low-dimensional subspace in Euclidean space 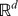.

Under the above assumption, the space 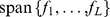, consisting of all linear combinations of functions 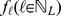, is a low-dimensional subspace in 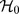. Let 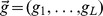. Denote an operator 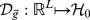,
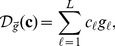
(4)where 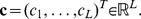 Let 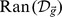 be the range of 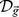 and 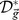 be the adjoint operator of 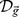. Then, 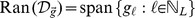. It is easy to see that 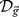 is a compact operator, and the dimension of 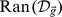 is finite and determined by the number of nonzero singular values of the operator 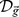. In order to enforce 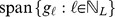 lying in a low-dimensional subspace in 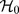, we can choose the following regularization term 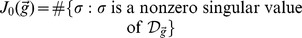, which equals to the number of nonzero eigenvalues of 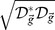, and regularizes the dimension of 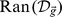. However, this regularization term is difficult to calculate as it is both nonconvex and nonsmooth. Motivated by the theory of compressed sensing and matrix completion [22], [23], we use a convex relaxation of 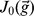 by taking the 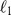 norm of all eigenvalues of 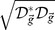 as the regularization term, that is,
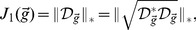
(5)where 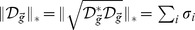 with 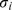 being the 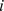-th singular value of 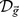.

We prove Theorem 1 in Material S1, which shows that the regularization term 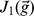 is convex, and can be rewritten as 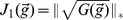, where 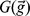 is an 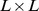 square matrix with the 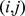 entry being 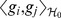, the inner product between 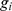 and 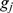 in 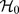.

#### Theorem 1

*Let* 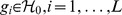 *and* 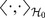 *be the inner product in* 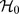 *. The operator* 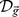 *is defined by (4). Then*

1. *for any* 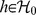, *we have* 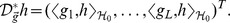

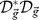
 *is a linear operator from* 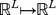 *and* 
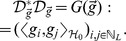

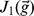
 *is convex. That is, for any* 
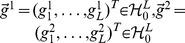
 *and* 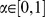, *we have* 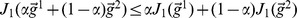.

Based on the above formulation and using least square error for the data fitting term, we therefore propose to learn gene expression programs in Hilbert space 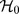 by minimizing the following objective function
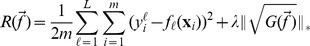
(6)where 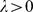 is a regularization parameter. We will refer to this model as *nonlinear low-rank model*.

#### Linear case

Next we will show that model (6) can be viewed as a generalization of the low-rank model (3) from linear setting to nonlinear setting. We assume that each target 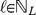 is well described by a linear function defined, for every 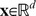, as 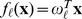, where 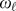 is a fixed vector of coefficients. As these linear functions are uniquely determined by those coefficients 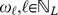, we can define the inner product for linear functions as
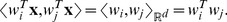
(7)That is, we have implicitly chosen the Hilbert space 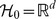. Denote 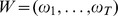. Using (7), we have 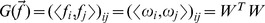. Note that 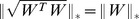. Therefore, (6) can be reformulated as
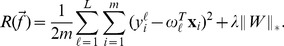
(8)


#### Kernel case

Reproducing kernel Hilbert space 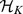 is widely used in statistical inference and machine learning [24]–[26]. It is associated with a Mercer kernel 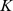 which is a continuous, symmetric and positive semidefinite function [27]. We denote its inner product as 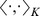. The reproducing property of 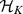 states that 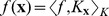, for all 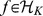.

The nonlinear low-rank model (6) can be much simplified when 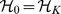. We prove the following representer theorem in Material S1.

#### Theorem 2

*Given a data set* 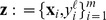 *, then the minimizer*

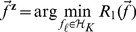
(9)
*exists and each component* 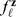 *takes the following form*

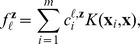

*where* 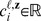 *for* 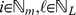.

As a consequence, the minimizer of (6) exists, and each component of 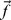 lies in the finite dimensional space spanned by 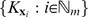, where 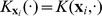. More specifically, we can show that the solution to the nonlinear low-rank model (6) is
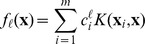
for each 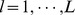, where 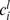
's are the coefficients. Furthermore, it can be shown that the coefficients are determined as the optimal solution that minimizes the following convex function
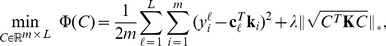
(10)where 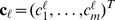 is a column vector, 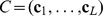 is an 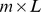 matrix, and 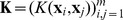 is an 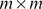 with the 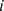-th column denoted by 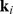. The problem (10) is of finite dimension, and next we describe an algorithm to solve it.

### Algorithms

In this section, we derive computational algorithms to solve low-rank regularized linear model (3) and nonlinear model (10).

#### Low-rank regularized linear model

Decompose the objective function (3), 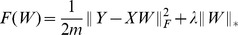 into two parts with 
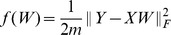
 and 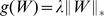. The first component 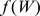 is both convex and differentiable, and 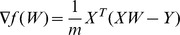. However, the second component 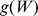 is not differentiable, although it is still convex.

Define 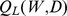 to be
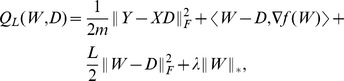
(11)where 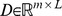 is a given matrix and 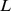 is a positive scalar. 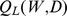 can be viewed as an approximation of 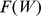 around 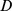, and the approximation is accurate when 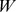 is sufficiently close to 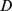. Although it is still a non-differentiable function of 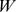, there exists a unique minimizer of 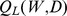 for a given 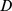, and the solution can be written down explicitly. Denote the minimizer of 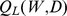 for a given 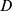 by 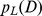. Next we write down an explicit formula for 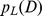.

Given a matrix 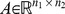 of rank 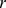, denote the singular value decomposition of 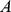 by 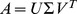, where 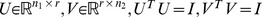, and 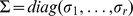 with 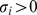 being singular values. For any 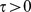, define the following soft-thresholding operator of matrix 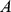


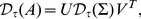
(12)where 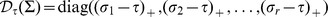 with 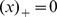 if 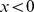 and 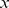 otherwise. With this definition, it can be shown that [17]

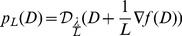
(13)


Using the above-mentioned notations and definitions, a Nesterov's algorithm [28] can be derived to solve the low-rank regularized linear model (3). The detailed steps are shown in Algorithm 1.

#### Algorithm 1

Nesterov's algorithm for solving (3) with backtracking Initialize 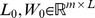. Set 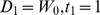


### repeat

Step k, 1) Find the smallest nonnegative integers 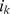 such that with 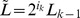


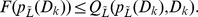



Step k, 2) Set 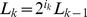 and compute
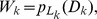


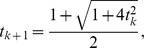


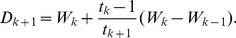



### until

Convergence

#### Low-rank regularized nonlinear model

We first convert the problem (10) into a more compact form by changing the optimization variables. Then we derive an algorithm to solve the problem based on the Nesterov's method [29], [30]. Note that 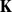 is symmetric and positive semidefinite, so is its square root 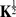. Denote the 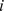-th column of 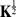 by 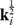, i.e., 
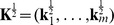
. Let 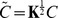 and write 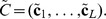 Then 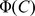 in equation (10) can be rewritten as a function of 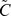


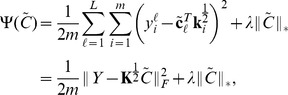
(14)where 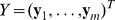 with 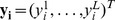. Thus finding a solution 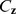 of equation (10) is equivalent to identifying,
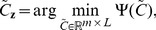
(15)followed by setting 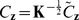 where 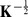 is the (pseudo) inverse of 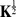 when 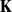 is (not) invertible.

Similar to the linear case, we first decompose 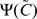 into two parts with 
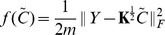
 and 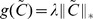. 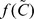 is differentiable and 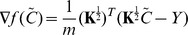.

Define 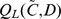 as the following form,
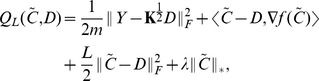
where 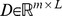 is a given matrix and 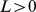.

The unique minimizer of 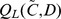 is denoted by 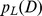, and we apply soft-thresholding operator (12) to give the explicit form of 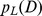,
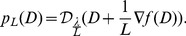
(16)With the above-mentioned notations and definitions, we derive Algorithm 3(′)@ to solve problem (10).

#### Algorithm 2

Nesterov's algorithm for solving (10) with backtracking Initialize 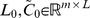. Set 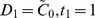


### repeat

Step k, 1) Find the smallest nonnegative integers 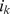 such that with 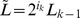


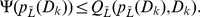



Step k, 2) Set 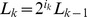 and compute
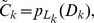


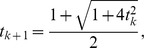


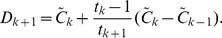



### until

Convergence

Return 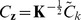


## Results

Next we test the performance of the low-rank regularization models (both linear and nonlinear) described above on three real biological datasets. We will compare the performance of our models to the Lasso model (2), which imposes a sparsity constraint within a linear regression framework, and the SiMoNe model [16], which models the modularity structure with a Gaussian graphical model framework. In each of the three experiments, we divide the data into training and test datasets. The models are trained based on training data, and the performance of the resulting models are then evaluated based on test data. We use root-mean-square error (RMSE) to measure the differences between values predicted by each model and the the values actually observed. The average RMSE over all target genes measured on the training and test data will be called training and testing error, respectively. The regularization parameter 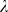 of our models is automatically tuned through ten-fold cross-validation based only on the training data, and is set to be the value that gives rise to the best cross-validation performance.

### Yeast gene expression data

We tested our models on a yeast gene expression dataset [31], which contains mRNA measurements of 2,355 genes of *Saccharomyces cerevisiae* responding to diverse environmental transitions including temperature shocks, amino acid starvation, hydrogen peroxide, etc. Overall the dataset contains microarray measurements of yeast genes in 173 environmental transitions (will be referred to as samples). The dataset was rescaled to make the expression values of each gene to be mean 0 and variance 1 across the 173 samples. We used a list of 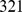 candidate regulators manually compiled in [15] based on biological annotations (including transcription factors and signaling molecules) as our regulator genes. We used this dataset to learn the regulatory relationship between these 321 regulators and the other 2,034 genes, which will be called targets. We benchmarked the performance of our and control models using ten-fold cross-validation. More specially, we randomly partitioned the 173 samples into 10 nonoverlapping subsets. Each model was trained using nine of the ten subsets, the regularization parameter 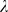 in each model was tuned via cross validation on 10 dimensional logarithmically spaced vector ranging between 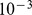 and 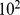. After choosing the lambda, the test performance of the learned model was then measured using the remaining subset. We used root-mean-square error (RMSE) to measure the differences between values predicted by each model and the the values actually observed. The average RMSE over all target genes measured on the training and test data will be called training and testing error, respectively.

**Figure 1:**
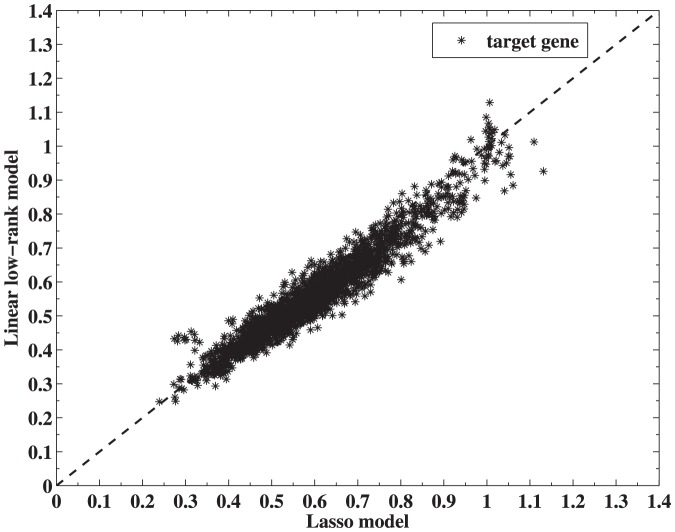
Comparison of the testing performance of the linear low-rank regularization model vs. Lasso on the yeast gene expression dataset. Each * indicates one target gene. X-axis represents the test RMSE of the Lasso model, whereas Y-axis represents the test RMSE of the linear low-rank model. The figure shows that the low-rank model yields lower testing error than Lasso for most target genes.

The training and test performance of four models - SiMoNe [16], linear low-rank (3), nonlinear low-rank (10), and Lasso (2), on the yeast gene expression dataset is summarized in Table 1. The linear low-rank model (3) reduces training error by 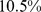 and testing error by 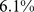 when compared to the Lasso model (2). The regression-based models, including ours and Lasso, significantly outperform SiMoNe in both training and test performance. If we look specifically at the prediction accuracy of each target gene, we note that 84% of the targets are predicted more accurately by the linear low-rank model than by Lasso (Figure 1). We used an ANOVA kernel to train the nonlinear low-rank model [32]. Although the training error of the nonlinear model is 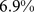 smaller than that of the linear low-rank model, the testing performance of the two models is similar. The optimal rank returned by the linear model is 78 and the one returned by the nonlinear model is 88, suggesting the existence of approximately 78–88 regulatory modules that are active in this dataset.

**Table 1:** Root-mean-squared error (RMSE) comparison among different models on the yeast gene expression data.

Model	Training error	Testing error
SiMoNe	—	1.0074±0.0650
Lasso	0.3897±0.0045	0.6124±0.0583
Linear low-rank	0.3488±0.0014	0.5750±0.0053
Nonlinear low-rank	0.3249±0.0063	0.5752±0.0054

“Lasso” represents model (2), “Linear low-rank” represents model (3), and “Nonlinear low-rank” represents model (6) with ANOVA kernel. SiMoNe is the model described by Chiquet et al. [16]. Both training and testing errors are measured in terms of RMSE averaged over all target genes. Shown here are mean ± standard deviation values of RMSEs in ten different runs.

### Human hematopoietic gene expression data

We also tested our models on a human hematopoietic gene expression dataset [33], which measures mRNA expression values of human genes during hematopoietic differentiation. The dataset contains expression profiles of 8,968 genes in 38 hematopoietic states with a total of 211 experiment conditions (will also be referred to as samples). We used a list of 523 candidate regulators manually complied in [33] based on biological annotations (including important transcriptional regulators or signalling molecules previously implicated in hematopoietic differentiation) as our regulator genes. Among the remaining non-regulator genes, we removed genes with low variance across the samples, and kept only the top 1000 genes with highest variance. These 1000 genes will be called target genes, and our goal is to learn the regulatory relationship between the 523 regulators and the 1000 target genes. We rescaled the expression of each gene (both regulators and targets) to be mean 0 and variance 1 across the samples. Similar to the yeast dataset, we benchmarked the performance of our and control models on this dataset using ten-fold cross-validation, and RMSE was used to measure both training and testing errors.

**Figure 2:**
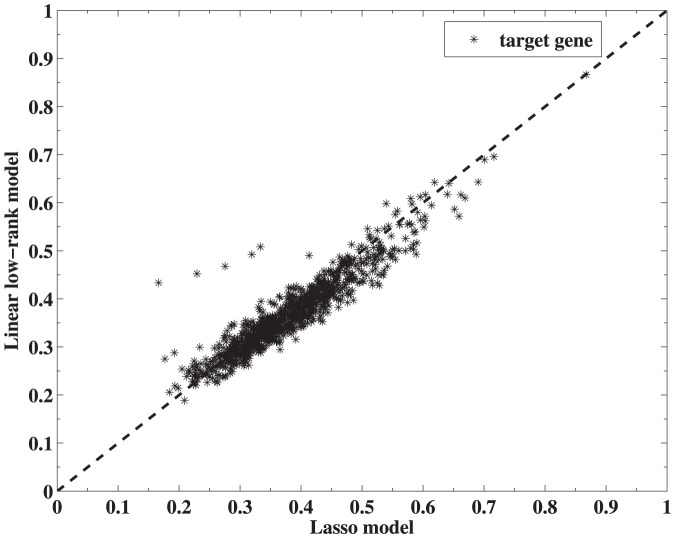
Comparison of the testing performance of the linear low-rank regularization model vs. Lasso on the human hematopoietic gene expression dataset. Each * indicates one target gene. X-axis represents the test RMSE of the Lasso model, whereas Y-axis represents the test RMSE of the linear low-rank model. The figure shows that the low-rank model yields lower testing error than Lasso for most target genes.

The training and test performance of four models - SiMoNe [16], linear low-rank (3), nonlinear low-rank (10), and Lasso (2), on the human hematopoietic gene expression dataset is summarized in Table 2. The linear low-rank model (3) reduces training error by 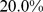 and testing error by 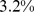 when compared to the Lasso model. Similar to the yeast dataset, the regression-based models outperform SiMoNe by a large margin. If we look specifically at the prediction accuracy of each target gene, we find that 70% of the targets are predicted more accurately by the linear low-rank model than by Lasso (Figure 2). Similar to the yeast dataset, we used an ANOVA kernel to train the nonlinear low-rank model. The training error of the nonlinear model is 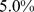 smaller than that of the linear low-rank model, but their testing performance is similar. The optimal rank returned by the linear model is 112 and the one returned by the nonlinear model is 109, suggesting that approximately 109–112 regulatory modules are active in this hematopoietic gene expression dataset.

**Table 2:** Root-mean-squared error (RMSE) comparison among different models on the human hematopoietic gene expression data.

Model	Training error	Testing error
SiMoNe	—	0.9987±0.0400
Lasso	0.2345±0.0024	0.3881±0.0265
Linear low-rank	0.1877±0.0030	0.3758±0.0265
Nonlinear low-rank	0.1783±0.0005	0.3767±0.0265

“Lasso” represents model (2), “Linear low-rank” represents model (3), and “Nonlinear low-rank” represents model (6) with ANOVA kernel. SiMoNe is the model described by Chiquet et al. [16]. Both training and testing errors are measured in terms of RMSE averaged over all target genes. Shown here are mean ± standard deviation values of RMSEs in ten different runs.

### Connectivity map data

The third dataset we have experimented with is the connectivity map data provided by Lamb et al. [2], which contains gene expression measurements of human cells responding to diverse treatments with chemical compounds and genetic reagents. The connectivity map data contains microarray measurements of human genes in thousands of profiles (will be referred to as samples). For regulator genes, we used 978 “landmark” genes determined by the connectivity map project as the set of genes that are most predictive of the expression of the other genes (Aravind Subramanian, personal communication). Among the remaining non-regulator genes, we used the top 10,000 genes with highest variances across samples as our target genes. We randomly selected 1000 samples from this dataset to benchmark the performance of our and other models (we were unable to use all samples due to computational constraints.) Our goal is to learn the regulatory relationship between the 10,000 target genes and the 978 landmark genes based on these 1000 samples.

We benchmarked the performance of our and control models using ten-fold cross-validation. More specifically, we randomly partitioned the samples into ten nonoverlapping subsets. Each model was trained using nine of the ten subsets, and the test performance of the learned model was then measured using the remaining subset. We used root-mean-square error (RMSE) to measure the differences between values predicted by each model and the the values actually observed. The average RMSE over all target genes measured on the training and test data will be called training and testing error, respectively.

We were unable to obtain SiMoNe results after hours of running the program, which might be due to the large number of genes contained in this dataset, and the fact that the inverse covariance matrix between these genes is too large to be handled by SiMoNe. So next we will focus on comparing the performance of our models to Lasso. The performance of the linear low-rank (3), nonlinear low-rank (10), and Lasso (2), on the connectivity map data is summarized in Table 3. The nonlinear low-rank model achieves the lowest testing error in this dataset. The testing performances of linear low-rank model and Lasso are similar, with the testing errors of both models 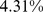 higher than the nonlinear low-rank model. If we compare the prediction performance of each target gene, 88% of the target genes are predicted more accurately by the nonlinear low-rank model than by Lasso.

**Table 3:** Root-mean-squared error (RMSE) comparison among different models on the connectivity map gene expression data.

Model	Training error	Testing error
Lasso	0.4943±0.0008	0.7077±0.0134
Linear low-rank	0.5157±0.0004	0.7000±0.0123
Nonlinear low-rank	0.4025±0.0005	0.6772±0.0125

“Lasso” represents model (2), “Linear low-rank” represents model (3), and “Nonlinear low-rank” represents model (6) with ANOVA kernel. Both training and testing errors are measured in terms of RMSE averaged over all target genes. Shown here are mean ± standard deviation values of RMSEs in ten different runs.

Our algorithms were implemented in Matlab and run on the platform of Intel Xeon E5-4617 - 2.9 GHz 1-Core CPU with 128 GB memory. The CPU times of running our algorithms on the three datasets are shown in Table 4. The time complexity of Algorithm 1 and 2 is mainly determined by the singular value decomposition step. Exact singular value decomposition of a 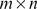 matrix has the time complexity of 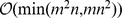. In our algorithms, 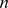 corresponds to the number of targets. However, 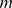 corresponds to the number of regulators in the linear model, and the number of samples in the nonlinear model. So when the number of samples is smaller than the number of regulators, the nonlinear model actually runs fasters than the linear model (See Table 4).

**Table 4:** CPU time of running the linear and nonlinear low-rank models.

Dataset	Linear low-rank (min)	Nonlinear low-rank (min)
Yeast gene expression	1.6	0.8
Human hematopoietic gene expression	1.0	0.4
Connectivity map gene expression	1067	869

## Discussion

Gene expression program learning is an important problem in both basic research as well as practical and applied settings of biotechnology development. In this paper, we formulate the gene expression program learning as a multi-target (more specifically 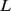-target) regression problem and use Tikhonov regularization to constrain the space of the L-target mapping. We propose a new form of regularization that constrains the number of independent connectivity patterns between regulator genes and target genes. We use the rank of the connectivity matrix from regulators to targets to represent the number of independent connectivity patterns, and approximate the rank of the matrix using its nuclear norm. The resulting low-rank regularization problem is convex, and we provide an efficient algorithm to find its globally optimal solution.

Previously, in gene expression program learning each target gene is usually treated separately. Because the expression of many genes are highly correlated, it would be beneficial to learn their expression regulation jointly instead of separately. However, it was unclear before on how to model the regulatory relationship from regulators to target genes jointly such that the resulting model is both computationally efficient and able to take the constraints between targets into account. The low-rank regularization provides an effective and yet computationally efficient framework for considering all target genes simultaneously. Experiments on two real gene expression datasets demonstrate that the low-rank model outperforms the Lasso model, one of the most widely used regularization method in gene expression program learning, in terms of prediction accuracy in both datasets.

We showed that the low-rank model can also be generalized to nonlinear settings, where we constrain the dimension of the hypothesis space of the 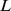-target regression function. We proved that the resulting problem is still convex and derived an efficient algorithm to find its globally optimal solution. We tested the nonlinear low-rank model on the gene expression datasets. The nonlinear low-rank model produces better testing performance than the linear low-rank model in some datasets, but is comparable to the linear model in other datasets. The lack of improvement comparing to the linear one in some datasets might be due to a) the fact that the number of samples used in these two datasets might be too small to fit a more complex model, and b) the kernel we have tried (ANOVA, Gaussian, and polynomial) might not be a good fit for the gene expression program learning. We expect that the nonlinear model will improve when a larger number of samples become available. So finding or designing the right kernel specifically for gene expression program learning will be the key to improving the nonlinear model.

The two forms of regularization, low-rank and sparsity, described in this paper are complementary to each other, considering two different aspects of gene regulation. A future direction is to combine these two regularizations into a single framework to constrain the connectivity matrix to be simultaneously sparse and low rank.

## Supporting Information

Material S1 For proving Theorems 1 and 2.
(PDF)
