# Correction: Low-Rank Regularization for Learning Gene Expression Programs

**DOI:** 10.1371/annotation/ab5352a4-6b6f-44e6-8ab5-6644794bc247

**Published:** 2014-01-13

**Authors:** Guibo Ye, Mengfan Tang, Jian-Feng Cai, Qing Nie, Xiaohui Xie

The Funding statement is incorrect. The Funding statement should read: 

"This work was partially supported by NIH P50GM76516 (to XX and QN), NSF DMS-1161621 (to QN) and NSF DBI-0846218 (to XX). The funders had no role in study design, data collection and analysis, decision to publish, or preparation of the manuscript."

