# Supplementary material for: Low-Rank Regularization for Learning Gene Expression Programs
Source: PLoS One. 2013 Dec 17;8(12):e82146. doi: 10.1371/journal.pone.0082146 (PMC3866120; doi:10.1371/journal.pone.0082146)
Supplement: Material S1 — For proving Theorems 1 and 2. (PDF) [file pone.0082146.s001.pdf]

Supplementary Information

Convexity of $J_1(\vec{g})$ and its reformulation

The following theorem gives us explicit formulas of operators $\mathcal{D}_{\vec{g}}^*$ and $\mathcal{D}_{\vec{g}}^*\mathcal{D}_{\vec{g}}$, and proves that $J_1(\vec{g})$ is convex.

Theorem 1. *Let $g_i \in \mathcal{H}_0, i = 1, \ldots, L$ and $\langle \cdot, \cdot \rangle_{\mathcal{H}_0}$ be the inner product in $\mathcal{H}_0$. The operator $\mathcal{D}_{\vec{g}}$ is defined by (4). Then*

(1) for any $h \in \mathcal{H}_0$, we have $\mathcal{D}_{\vec{g}}^* h = (\langle g_1, h\rangle_{\mathcal{H}_0}, \ldots, \langle g_L, h\rangle_{\mathcal{H}_0})^T$.

(2) $\mathcal{D}_{\vec{g}}^*\mathcal{D}_{\vec{g}}$ is a linear operator from $\mathbb{R}^L \mapsto \mathbb{R}^L$ and $\mathcal{D}_{\vec{g}}^*\mathcal{D}_{\vec{g}} = G(\vec{g}) := (\langle g_i, g_j\rangle_{\mathcal{H}_0})_{i,j\in\mathbb{N}_L}$.

(3) $J_1(\vec{g})$ is convex. That is, for any $\vec{g}^1 = (g_1^1, \ldots, g_L^1)^T \in \mathcal{H}_0^L, \vec{g}^2 = (g_1^2, \ldots, g_L^2)^T \in \mathcal{H}_0^L$ and $\alpha \in [0, 1]$, we have $J_1(\alpha\vec{g}^1 + (1-\alpha)\vec{g}^2) \le \alpha J_1(\vec{g}^1) + (1-\alpha)J_1(\vec{g}^2)$.

Proof. (1) According to the definition of adjoint operator, we have $\langle \mathcal{D}_{\vec{g}}^* h, \mathbf{c}\rangle_{\mathbb{R}^L} = \langle \mathcal{D}_{\vec{g}}\mathbf{c}, h\rangle_{\mathcal{H}_0}$. On the other hand, by the definition of $\mathcal{D}_{\vec{g}}$, we have $\langle \mathcal{D}_{\vec{g}}\mathbf{c}, h\rangle_{\mathcal{H}_0} = \langle \sum_{i=1}^L c_i g_i, h\rangle_{\mathcal{H}_0} = \sum_{i=1}^L c_i\langle g_i, h\rangle_{\mathcal{H}_0}$. Denote $\mathbf{d} = (\langle g_1, h\rangle_{\mathcal{H}_0}, \ldots, \langle g_L, h\rangle_{\mathcal{H}_0})^T$. Then $\langle \mathcal{D}_{\vec{g}}^* h, \mathbf{c}\rangle_{\mathbb{R}^L} = \sum_{i=1}^T c_i\langle g_i, h\rangle_{\mathcal{H}_0} = \langle \mathbf{c}, \mathbf{d}\rangle_{\mathbb{R}^L}$ which implies $\mathcal{D}_{\vec{g}}^* h = \mathbf{d} = (\langle g_1, h\rangle_{\mathcal{H}_0}, \ldots, \langle g_L, h\rangle_{\mathcal{H}_0})^T$.

(2) Note that $\mathcal{D}_{\vec{g}}$ is a map from $\mathbb{R}^L \mapsto \mathcal{H}_0$. Thus $\mathcal{D}_{\vec{g}}^*\mathcal{D}_{\vec{g}}$ is a linear operator from $\mathbb{R}^L \mapsto \mathbb{R}^L$. For any $\mathbf{c} \in \mathbb{R}^L$, we have

$$
\begin{aligned}
(\mathcal{D}_{\vec{g}}^*\mathcal{D}_{\vec{g}}\mathbf{c})_i &= (\mathcal{D}^*(\sum_j c_j g_j))_i = \sum_j c_j(\mathcal{D}_{\vec{g}}^* g_j)_i \\
&= \sum_j c_j\langle g_j, g_i\rangle_{\mathcal{H}_0} = \sum_j \langle g_i, g_j\rangle_{\mathcal{H}_0} c_j.
\end{aligned}
$$

That is, $\mathcal{D}_{\vec{g}}^*\mathcal{D}_{\vec{g}}\mathbf{c} = G(\vec{g})\mathbf{c}$. Therefore, $\mathcal{D}_{\vec{g}}^*\mathcal{D}_{\vec{g}} = G(\vec{g}) = (\langle g_i, g_j\rangle_{\mathcal{H}_0})_{i,j\in\mathbb{N}_L}$.

(3) According to the definition of $\mathcal{D}_{\vec{g}}$ by (4), $\mathcal{D}_{\alpha\vec{g}^1+(1-\alpha)\vec{g}^2} = \alpha\mathcal{D}_{\vec{g}^1} + (1-\alpha)\mathcal{D}_{\vec{g}^2}$. Therefore,

$$
\begin{aligned}
& J_1(\alpha\vec{g}^1 + (1-\alpha)\vec{g}^2) \\
= \;& \|\mathcal{D}_{\alpha\vec{g}^1+(1-\alpha)\vec{g}^2}\|_* = \|\alpha\mathcal{D}_{\vec{g}^1} + (1-\alpha)\mathcal{D}_{\vec{g}^2}\|_* \\
\le \;& \alpha\|\mathcal{D}_{\vec{g}^1}\|_* + (1-\alpha)\|\mathcal{D}_{\vec{g}^2}\|_* \\
= \;& \alpha J_1(\vec{g}^1) + (1-\alpha)J_1(\vec{g}^2).
\end{aligned}
$$

□

Representer theorem

Theorem 2. *Given a data set $\mathbf{z} := \{\mathbf{x}_i, y_i^\ell\}_{i=1}^m$, then the minimizer*

$$\vec{f}^{\mathbf{z}} = \arg\min_{f_\ell\in\mathcal{H}_K} R_1(\vec{f}) \tag{16}$$

exists and each component $f_\ell^{\mathbf{z}}$ takes the following form

$$f_\ell^{\mathbf{z}} = \sum_{i=1}^m c_i^{\ell,\mathbf{z}} K(\mathbf{x}_i, \mathbf{x}),$$

where $c_i^{\ell,\mathbf{z}} \in \mathbb{R}$ for $i \in \mathbb{N}_m, \ell \in \mathbb{N}_L$.

Proof. The existence follows from the convexity of the loss function and regularization term $J_1(\vec{g})$ shown by Theorem 1.

Since $f_\ell^{\mathbf{z}} \in \mathcal{H}_K$ for each $\ell \in \mathbb{N}_L$, we can write $f_\ell^{\mathbf{z}}$ as $f_\ell^{\mathbf{z}} = f_\ell^{\mathbf{z},\|} + f_\ell^{\mathbf{z},\perp}, \ell \in \mathbb{N}_L$ where $f_\ell^{\mathbf{z},\|}$ is in the span of $\{K_{\mathbf{x}_i} : i \in \mathbb{N}_m\}$ and $g_\ell^{\mathbf{z},\perp}$ is a function in the orthogonal complement. It is easy to see that $\langle f_i^{\mathbf{z},\|}, f_j^{\mathbf{z},\perp}\rangle_K = 0, \forall i,j \in \mathbb{N}_L$. The reproducing property yields $f_\ell^{\mathbf{z}}(\mathbf{x}_i) = f_\ell^{\mathbf{z},\|}(\mathbf{x}_i)$ for all $\mathbf{x}_i$. So the function $f_\ell^{\mathbf{z},\perp}$ do not have an effect on the data fitting term.

Let $G^{\mathbf{z},\|}(\vec{f}) = (\langle f_i^{\mathbf{z},\|}, f_j^{\mathbf{z},\|}\rangle_K)_{i,j=1}^L$, $G^{\mathbf{z},\perp}(\vec{f}) = (\langle f_i^{\mathbf{z},\perp}, f_j^{\mathbf{z},\perp}\rangle_K)_{i,j=1}^L$ and $G^{\mathbf{z}}(\vec{f}) = (\langle f_i^{\mathbf{z}}, f_j^{\mathbf{z}}\rangle_K)_{i,j=1}^L$. It is easy to see that if we can prove $\|\sqrt{G^{\mathbf{z}}(\vec{f})}\|_* \geq \|\sqrt{G^{\mathbf{z},\|}(\vec{f})}\|_*$, then the theorem holds. Note that $\langle f_i^{\mathbf{z}}, f_j^{\mathbf{z}}\rangle_K = \langle f_i^{\mathbf{z},\|}, f_j^{\mathbf{z},\|}\rangle_K + \langle f_i^{\mathbf{z},\perp}, f_j^{\mathbf{z},\perp}\rangle_K$ by using the fact $\langle f_i^{\mathbf{z},\|}, f_j^{\mathbf{z},\perp}\rangle_K = 0$ for all $i,j = 1,\ldots,L$. Therefore, $G^{\mathbf{z}}(\vec{f}) = G^{\mathbf{z},\|}(\vec{f}) + G^{\mathbf{z},\perp}(\vec{f})$ and $G^{\mathbf{z}}(\vec{f}), G^{\mathbf{z},\|}(\vec{f}), G^{\mathbf{z},\perp}(\vec{f})$ are all positive semidefinite. Let $\lambda_i(G^{\mathbf{z}}(\vec{f}))$ be the i-th eigenvalue of $G^{\mathbf{z}}(\vec{f})$ and $\lambda_i(G^{\mathbf{z},\|}(\vec{f}))$ be the i-th eigenvalue of $G^{\mathbf{z},\|}(\vec{f})$. If we can show that $\lambda_i(G^{\mathbf{z}}(\vec{f})) \geq \lambda_i(G^{\mathbf{z},\|}(\vec{f}))$ for any $i = 1,\ldots,L$, then $\sqrt{\lambda_i(G^{\mathbf{z}}(\vec{f}))} \geq \sqrt{\lambda_i(G^{\mathbf{z},\|}(\vec{f}))}$ for any $i = 1,\ldots,L$ which implies $\|\sqrt{G^{\mathbf{z}}(\vec{f})}\|_* \geq \|\sqrt{G^{\mathbf{z},\|}(\vec{f})}\|_*$. In fact,

$$\begin{aligned}\lambda_1(G^{\mathbf{z}}(\vec{f})) &= \max_{\|\mathbf{a}\|=1} \mathbf{a}^T(G^{\mathbf{z},\|}(\vec{f}) + G^{\mathbf{z},\perp}(\vec{f}))\mathbf{a} \\ &\geq \max_{\|\mathbf{a}\|=1} \mathbf{a}^T G^{\mathbf{z},\|}(\vec{f})\mathbf{a} = \lambda_1(G^{\mathbf{z},\|}(\vec{f})).\end{aligned}$$

Let $\mathbf{u}_i$ be the i-th eigenvector of $G^{\mathbf{z}}$ and S_i be any i-dimensional subspace of $\mathbb{R}^L$. Then for $2 \leq i \leq L$,

$$\begin{aligned}\lambda_i(G^{\mathbf{z}}(\vec{f})) &= \max_{\mathbf{a}\perp\mathbf{u}_j, 1\leq j\leq i-1, \|\mathbf{a}\|=1} \mathbf{a}^T(G^{\mathbf{z},\|} + G^{\mathbf{z},\perp})\mathbf{a} \\ &\geq \max_{\mathbf{a}\perp\mathbf{u}_j, 1\leq j\leq i-1, \|\mathbf{a}\|=1} \mathbf{a}^T G^{\mathbf{z},\|}(\vec{f})\mathbf{a} \\ &\geq \min_{S_{i-1}} \max_{\mathbf{a}\in S_{i-1}, \|\mathbf{a}\|=1} \mathbf{a}^T G^{\mathbf{z},\|}(\vec{f})\mathbf{a} \\ &= \lambda_i(G^{\mathbf{z},\|}(\vec{f})).\end{aligned}$$

The last equality follows from Courant-Fischer-Weyl min-max principle [34]. □

Low-rank plus sparsity in linear and nonlinear cases

We can also combine sparsity and low-rank regularization. We assume the coefficient matrix W may admit a low-rank structure as well as an entry-wise sparse structure. More specifically, let $W = \mathbf{L} + \mathbf{S}$, where $\mathbf{L}$ is a low-rank matrix modeling the relationship between different target genes and $\mathbf{S}$ is a sparse matrix modeling the distinct feature of each target gene. Under this assumption, we propose the *low-rank plus sparsity* (abbreviated as $L + S$) model to learn gene expression programs:

$$\min_{L,S\in\mathbb{R}^{p\times L}} \frac{1}{2m}\|Y - X(\mathbf{L}+\mathbf{S})\|_F^2 + \lambda_1\|\mathbf{L}\|_* + \lambda_2\|\mathbf{S}\|_1. \tag{17}$$

Similar to the linear setting, the mappings $f_\ell, \ell \in \mathbb{N}_L$ might not exactly lie in a low-dimensional subspace of $\mathcal{H}_0$ since each mapping might also have its own unique characteristics besides the shared common features. In this case, we assume that each target mapping can be written in the form $f_\ell = g_\ell + h_\ell, \ell \in \mathbb{N}_L$, where $g_\ell \in \mathcal{H}_0, h_\ell \in \mathcal{H}_\ell$, $\mathcal{H}_0$ and $\mathcal{H}_\ell$ are Hilbert spaces and could be different. Different target mappings are related through g_ℓ's, which lie in a common subspace in Hilbert space $\mathcal{H}_0$, whereas h_ℓ represents the discriminative characteristic of each individual mapping. To regularize $g_\ell, \ell \in \mathbb{N}_L$, we

use $\|G(\vec{g})\|_*$ by the same argument as in model (6). To regularize h_ℓ, we assume that h_ℓ's are linear with sparse loading in a known finite dimensional feature space span$\{\phi_i, i = 1, \ldots, N\}$, then we can choose the regularization term for h_ℓ to be $\|W\|_1$, where $W = (w_1, \ldots, w_L)^T = (w_{\ell i})_{\ell i}$ with w_ℓ being the weight for function h_ℓ, and $\|W\|_1 = \sum_{i=1}^N \sum_{\ell=1}^L |w_{\ell i}|$. Then, the L-target mapping function $\vec{f}_{\mathbf{z}}$ can be estimated via $\vec{f}_{\mathbf{z}} = \vec{g}_{\mathbf{z}} + \sum_{j=1}^N \omega_{\ell j}^{\mathbf{z}} \phi_j$ with $(\vec{g}_{\mathbf{z}}, W_{\mathbf{z}})$ being the solution of the following minimization problem

$$\begin{aligned} & R(\vec{g}, W) \\ = \; & \frac{1}{2m} \sum_{\ell=1}^L \sum_{i=1}^m \left(y_i^\ell - \left(g_\ell(\mathbf{x}_i) + \sum_{j=1}^N w_{\ell j} \phi_j(\mathbf{x}_i) \right) \right)^2 \\ & + \lambda_1 \| \sqrt{G(\vec{g})} \|_* + \lambda_2 \|W\|_1, \end{aligned} \tag{18}$$

where $\lambda_1, \lambda_2 > 0$ are two regularization parameters. We will refer to this model as *low-rank plus sparsity Hilbert space model*. Using a similar argument, we have the representer theorem for model (18).

Theorem 3. *Given a data set* $\mathbf{z} := \{\mathbf{x}_i, y_i^\ell\}_{i=1}^m$, *then the minimizer*

$$(\vec{f^{\mathbf{z}}}, W_{\mathbf{z}}) = \arg \min_{f_\ell \in \mathcal{H}_K, W \in \mathbb{R}^{L \times N}} R(\vec{f}, W) \tag{19}$$

exists and each component $f_\ell^{\mathbf{z}}$ *takes the following form*

$$f_\ell^{\mathbf{z}} = \sum_{i=1}^m c_i^{\ell, \mathbf{z}} K(\mathbf{x}_i, \mathbf{x}) + \sum_{j=1}^N \omega_{\ell j}^{\mathbf{z}} \phi_j(\mathbf{x}),$$

where $c_i^{\ell,\mathbf{z}}, \omega_{\ell,j}^{\mathbf{z}} \in \mathbb{R}$ *for* $i \in \mathbb{N}_m, \ell \in \mathbb{N}_L, j \in \mathbb{N}_N$.

For the model (18), we have a similar representer theorem and the problem is equivalent to solving

$$(C_{\mathbf{z}}, W_{\mathbf{z}}) = \arg \min_{\substack{C \in \mathbb{R}^{m \times L} \\ W \in \mathbb{R}^{N \times L}}} \Phi(C, W), \tag{20}$$

where $\Phi(C, W) = \frac{1}{2m} \sum_{\ell=1}^L \sum_{i=1}^m (y_i^\ell - (\mathbf{c}_\ell^T \mathbf{k}_i + \sum_{j=1}^N w_{\ell j} \phi_j(\mathbf{x}_i)))^2 + \lambda_1 \| \sqrt{C^T \mathbf{K} C} \|_* + \lambda_2 \|W\|_1$.

Algorithm

For the model (19), using the similar argument, finding a solution $(C_{\mathbf{z}}, W_{\mathbf{z}})$ of minimization (20) is equivalent to identifying

$$(\widetilde{C}_{\mathbf{z}}, W_{\mathbf{z}}) = \arg\min \Psi_2(\widetilde{C}, W), \tag{21}$$

followed by setting $C_{\mathbf{z}} = \mathbf{K}^{-\frac{1}{2}} \widetilde{C}_{\mathbf{z}}$, where

$$\begin{aligned} & \Psi_2(\widetilde{C}, W) \\ = \; & \frac{1}{2m} \|Y - (\mathbf{K}^{\frac{1}{2}} \widetilde{C} + \hat{\Phi} W)\|_F^2 + \lambda_1 \|\widetilde{C}\|_* + \lambda_2 \|W\|_1 \end{aligned} \tag{22}$$

with $\hat{\Phi} = (\phi_j(\mathbf{x}_i))_{ij}$ and $W = (\omega_1, \ldots, \omega_L)$.

Now we derive the algorithm for (21). We decompose $\Psi_2(\widetilde{C}, W)$ into two parts with $f(\widetilde{C}, W) = \frac{1}{2m} \|Y - (\mathbf{K}^{\frac{1}{2}} \widetilde{C} + \hat{\Phi} W)\|_F^2$ and $g(\widetilde{C}, W) = \lambda_1 \|\widetilde{C}\|_* + \lambda_2 \|W\|_1$. Then

$$\nabla_{\widetilde{C}} f(\widetilde{C}, W) = \frac{1}{m} \mathbf{K}^{\frac{1}{2}} (\mathbf{K}^{\frac{1}{2}} \widetilde{C} + \hat{\Phi} W - Y),$$

$$\nabla_W f(\widetilde{C}, W) = \frac{1}{m}\hat{\Phi}^T(\mathbf{K}^{\frac{1}{2}}\widetilde{C} + \hat{\Phi}W - Y),$$

and

$$\begin{aligned} & Q_L(\widetilde{C}, W, D, V) \\ = \quad & \frac{1}{2m}\|Y - (\mathbf{K}^{\frac{1}{2}}D + \hat{\Phi}V)\|_F^2 + \langle \widetilde{C} - D, \nabla_{\widetilde{C}} f(\widetilde{C}, W)\rangle \\ & + \langle W - V, \nabla_W f(\widetilde{C}, W)\rangle + \frac{L}{2}\|\widetilde{C} - D\|_F^2 \\ & + \frac{L}{2}\|W - V\|_F^2 \lambda_1 \|\widetilde{C}\|_* + \lambda_2\|W\|_1 \end{aligned}$$

Let $\mathcal{T}_\lambda$ be a soft thresholding operator defined on vector space and satisfying

$$\mathcal{T}_\lambda(W) = (t_\lambda(W_{ij}))_{ij}, \forall W \in \mathbb{R}^{N \times L}, \tag{23}$$

where

$$t_\lambda(W_{ij}) = \operatorname{sgn}(W_{ij}) \max\{0, |W_{ij}| - \lambda\}.$$

Then

$$p_L(D, V) = \begin{pmatrix} \mathcal{D}_{\frac{\lambda_1}{L}}(D + \frac{1}{L}\nabla_D f(D, V)) \\ \mathcal{T}_{\frac{\lambda_2}{L}}(V + \frac{1}{L}\nabla_V f(D, V)) \end{pmatrix} \tag{24}$$

Using these notations, we can get the explicit algorithm for (21).
